# Age-specific prevalence of hepatitis B virus infection in young pregnant women, Hong Kong Special Administrative Region of China

**DOI:** 10.2471/BLT.13.133413

**Published:** 2014-09-03

**Authors:** Terence T Lao, Daljit S Sahota, Lai-Wa Law, Yvonne KY Cheng, Tak-Yeung Leung

**Affiliations:** a Department of Obstetrics and Gynaecology, Chinese University of Hong Kong, Prince of Wales Hospital, Ngan Shing Street, Shatin, New Territories, Hong Kong Special Administrative Region, China.

## Abstract

**Objective:**

To investigate the age-specific prevalence of hepatitis B virus (HBV) infection in young pregnant women in Hong Kong Special Administrative Region (SAR), China, and to determine whether an increase in prevalence occurs during adolescence.

**Methods:**

HBV prevalence was quantified using data from routine antenatal screening for hepatitis B surface antigen (HBsAg) in 10 808 women aged 25 years or younger born in Hong Kong SAR and managed at a single hospital between 1998 and 2011. The effect on prevalence of maternal age, parity and birth before or after HBV vaccine availability in 1984 was assessed, using Spearman’s correlation and multiple logistic regression analysis.

**Findings:**

Overall, 7.5% of women were HBsAg-positive. The prevalence ranged from 2.3% to 8.4% in those aged ≤ 16 and 23 years, respectively. Women born in or after 1984 and those younger than 18 years of age were less likely to be HBsAg-positive (odds ratio, OR: 0.679; 95% confidence interval, CI: 0.578–0.797) and (OR: 0.311; 95% CI: 0.160–0.604), respectively. For women born before 1984, there was no association between HBsAg carriage and being younger than 18 years of age (OR: 0.60; 95% CI: 0.262–1.370) Logistic regression analysis showed that the prevalence of HBsAg carriage was influenced more by the woman being 18 years old or older (adjusted OR, aOR: 2.80; 95% CI: 1.46–5.47) than being born before 1984 (aOR: 1.42; 95% CI: 1.21–1.67).

**Conclusion:**

Immunity to HBV in young pregnant women who had been vaccinated as neonates decreased in late adolescence.

## Résumé

**Objectif:**

Étudier la prévalence selon l'âge de l'infection par le virus de l'hépatite B (VHB) chez les jeunes femmes enceintes dans la Région administrative spéciale (RAS) de Hong Kong de Chine, et déterminer si une augmentation de la prévalence a lieu pendant l'adolescence.

**Méthodes:**

La prévalence du VHB a été quantifiée en utilisant les données des dépistages prénataux de routine pour détecter l'antigène de surface de l'hépatite B (HBsAG) chez 10 808 femmes âgées de moins de 25 ans nées dans la RAS de Hong Kong et suivies dans un seul hôpital entre 1998 et 2011. Les effets sur la prévalence de l'âge maternel, de la parité et de la naissance avant ou après la disponibilité du vaccin anti-VHB en 1984 ont été évalués en utilisant la corrélation de Spearman et une analyse de régression logistique multiple.

**Résultats:**

Globalement, 7,5% des femmes étaient positives pour l'antigène HBsAg. La prévalence était comprise entre 2,3% et 8,4% chez les femmes âgées de ≤ 16 et 23 ans, respectivement. Les femmes nées en 1984 ou après 1984 et les femmes âgées de moins de 18 ans étaient moins susceptibles d'être positives pour l'antigène HBsAg (rapport des cotes, RC: 0,679; intervalle de confiance à 95%, IC 95%: 0,578–0,797) et (RC: 0,311; IC 95%: 0,160–0,604), respectivement. Pour les femmes nées avant 1984, il n'y avait pas d'association entre être dépistée positive pour l'antigène HBsAG et avoir moins de 18 ans (RC: 0,60; IC 95%: 0,262–1,370). L'analyse de régression logistique a montré que la prévalence de la présence de l'antigène HBsAg chez les femmes était davantage influencée par le fait d'avoir 18 ans ou plus (RC ajusté, RCa: 2,80; IC 95%: 1,46–5,47) que par le fait d'être née avant 1984 (RCa: 1,42; IC 95%: 1,21–1,67).

**Conclusion:**

L'immunité contre le VHB chez les jeunes femmes enceintes qui ont été vaccinées dès leur naissance a diminué à la fin de l'adolescence.

## Resumen

**Objetivo:**

Investigar la prevalencia por edad de la infección por el virus de la hepatitis B (VHB) en mujeres embarazadas jóvenes en Hong Kong, Región Administrativa Especial (SAR) de China, y determinar si durante la adolescencia se produce un aumento de la prevalencia.

**Métodos:**

La prevalencia del VHB se cuantificó a partir de datos de las pruebas rutinarias de detección prenatal para el antígeno de superficie del virus de la hepatitis B (HBsAg) en 10 808 mujeres de 25 años o menores nacidas en Hong Kong SAR y gestionados en un solo hospital entre 1998 y 2011. Se evaluó el efecto de la edad de la madre, el número de veces que habían dado a luz y el nacimiento antes o después de la disponibilidad de la vacuna del VHB en 1984 sobre la prevalencia. Para ello se empleó la correlación de Spearman y análisis de regresión logística múltiple.

**Resultados:**

En general, el 7,5% de las mujeres dio resultados positivos para el HBsAg. La prevalencia varió del 2,3% al 8,4% en las mujeres de 16 años o mayores y 23 de años o mayores, respectivamente. Las mujeres nacidas en el año 1984 o más tarde, y las menores de 18 años de edad tenían menos probabilidad de dar positivo para el HBsAg (proporción de probabilidad, OR: 0,679; intervalo de confianza del 95%, IC: 0,578–0,797) y (OR: 0,311; 95% IC: 0,160–0,604), respectivamente. Para las mujeres nacidas antes de 1984, no hubo asociación entre ser portadoras de HBsAg y ser menores de 18 años de edad (OR: 0,60; 95% IC: 0,262–1,370). El análisis de regresión logística mostró que la prevalencia de las portadoras de HBsAg se vio más influida por tener 18 o más años de edad (OR ajustada, proporción de probabilidades ajustadas: 2,80; 95% IC: 1,46–5,47) que por haber nacido antes de 1984 (proporción de probabilidades ajustadas: 1,42; 95% IC: 1,21–1,67).

**Conclusión:**

La inmunidad frente al VHB en mujeres jóvenes embarazadas que habían sido vacunadas como recién nacidos disminuyó a finales de la adolescencia.

## ملخص

**الغرض:**

تحري معدل انتشار عدوى فيروس التهاب الكبد "باء" حسب الفئة العمرية بين النساء الحوامل صغار السن في منطقة هونغ كونغ الصينية الإدارية الخاصة وتحديد ما إذا كانت هناك زيادة في معدل الانتشار خلال سن المراهقة.

**الطريقة:**

تم تحديد مقدار انتشار فيروس التهاب الكبد "باء" باستخدام البيانات المستمدة من الفحص الروتيني السابق للولادة للمستضد السطحي لالتهاب الكبد "باء" (HBsAg) لدى 10808 امرأة في سن 25 عاماً أو أصغر ممن ولدن في منطقة هونغ كونغ الإدارية الخاصة وتلقين التدبير العلاجي في مستشفى واحد في الفترة من عام 1998 إلى عام 2011. وتم تقييم التأثير على معدل انتشار سن الأم والتكافؤ والولادة قبل توافر لقاح فيروس التهاب الكبد "باء" في عام 1984 أو بعده، باستخدام ارتباط سبيرمان وتحليل الارتداد اللوجيستي المتعدد.

**النتائج:**

بشكل عام، كانت نسبة 7.5 % من النساء إيجابيات للمستضد السطحي لالتهاب الكبد "باء". وتراوح معدل الانتشار من 2.3 % إلى 8.4 % بين النساء في سن 16 و23 سنة، على التوالي. وكانت احتمالية الإيجابية للمستضد السطحي لالتهاب الكبد "باء" أقل لدى النساء اللاتي ولدن في عام 1984 أو بعده والنساء دون سن 18 سنة (نسبة الاحتمال: 0.679؛ فاصل الثقة 95 %، فاصل الثقة: من 0.578 إلى 0.797) و(نسبة الاحتمال: 0.311؛ فاصل الثقة 95 %: من 0.160 إلى 0.604)، على التوالي. ولم يكن هناك ارتباط بين انتقال المستضد السطحي لالتهاب الكبد "باء" (HBsAg) والسن الذي هو دون 18 سنة لدى النساء اللاتي ولدن قبل عام 1984 (نسبة الاحتمال: 0.60؛ فاصل الثقة 95 %: من 0.262 إلى 1.370) وأظهر تحليل الارتداد اللوجيستي تأثر معدل انتشار انتقال المستضد السطحي لالتهاب الكبد "باء" (HBsAg) على نحو أكبر بسن المرأة التي تبلغ من العمر 18 سنة أو أكبر (نسبة الاحتمال المصححة: 2.80؛ فاصل الثقة 95 %: من 1.46 إلى 5.47) عنه بالميلاد قبل عام 1984 (نسبة الاحتمال المصححة: 1.42؛ فاصل الثقة 95 %: من 1.21 إلى 1.67).

**الاستنتاج:**

انخفضت المناعة ضد فيروس التهاب الكبد "باء" بين النساء الحوامل صغار السن اللاتي حصلن على التطعيم كمواليد في المراهقة المتأخرة.

## 摘要

**目的:**

调查中国香港特别行政区（SAR）年轻孕妇的特定年龄段乙型肝炎病毒（HBV）感染流行率，并确定青春期流行率是否有增加。

**方法:**

使用在1998年至2011年在单所医院进行管理的10808名25岁以下特区出生女性的乙型肝炎表面抗原（HBsAg）常规产前筛查数据量化HBV流行率。使用斯皮尔曼相关性和多元逻辑回归分析评估产妇年龄、胎次以及1984年乙型肝炎病毒疫苗上市前后出生等因素对发病率的影响。

**结果:**

总体而言，7.5%女性为HBsAg阳性。不超过16岁和不超过23岁女性发病率分别为2.3%至8.4%。在1984年或之后出生的女性以及未满18岁的女性HBsAg阳性可能性更低，分别为（优势比，OR：0.679；95%置信区间，CI：0.578-0.797）和（OR：0.311；95% CI：0.160-0.604）。对于在1984年之前出生的女性，在HBsAg携带和未满18岁（OR：0.60；95% CI：0.262-1.370）之间没有关联。逻辑回归分析显示，较之1984年之前出生（aOR：1.42；95% CI：1.21-1.67），女性年满18岁对HBsAg携带影响更大（调整的OR，aOR：2.80；95% CI：1.46-5.47）。

**结论:**

在新生儿期间接种疫苗的年轻孕妇在青春晚期乙肝病毒免疫力降低。

## Резюме

**Цель:**

Исследовать зависимость распространения вируса гепатита B (HBV) от возраста среди молодых беременных женщин в Специальном административном округе Гонконг, Китай, и определить, возрастает ли уровень распространения заболевания в подростковом возрасте.

**Методы:**

Количественные показатели распространения вируса гепатита B (HBV) были определены на основе данных, полученных в ходе планового предродового скринингового обследования на наличие поверхностного антигена гепатита B (HBsAg) среди 10 808 женщин в возрасте 25 лет или менее, рожденных в Специальном административном округе Гонконг, Китай, с 1998 по 2011 гг. Была выполнена оценка влияния на распространение вируса таких факторов, как возраст матери, количество родов, рождение до или после появления вакцины от гепатита B в 1984 году; для выполнения оценки использовались корреляция Спирмана и многочисленные методы регрессивного анализа.

**Результаты:**

Было обнаружено, что в общей сложности 7,5% женщин являются носителями поверхностного антигена вируса гепатита B. Уровень распространения варьировался от 2,3% до 8,4% в возрастных группах ≤ 16 лет и 23 лет соответственно. Женщины, рожденные в 1984 году и позднее, а также женщины младше 18 лет реже являются носителями поверхностного антигена вируса гепатита B (коэффициент вероятности: 0,679, 95%; доверительный интервал: 0,578–0,797) и (коэффициент вероятности: 0,311, 95%; доверительный интервал: 0,160–0,604) соответственно. Для женщин, рожденных до 1984 года, не было установлено связи между наличием поверхностного антигена вируса гепатита B и возрастом младше 18 лет (коэффициент вероятности: 0,60, 95%; доверительный интервал: 0,262–1,370). Метод логистического регрессивного анализа показал, что поверхностный антиген вируса гепатита B чаще распространен среди женщин 18 лет и старше (откорректированный коэффициент вероятности: 2,80, 95%; доверительный интервал: 1,46–5,47), чем среди рожденных до 1984 года (откорректированный коэффициент вероятности: 1,42, 95%; доверительный интервал: 1,21–1,67).

**Вывод:**

Иммунитет к вирусу гепатита B у молодых женщин, которые прошли вакцинацию в младенческом возрасте, снижается в позднем подростковом периоде.

## Introduction

It is generally held that vertical transmission is the major route of hepatitis B virus (HBV) infection in regions where the disease is endemic.^1^^–^^5^ The neonatal immunization programmes using immunoglobulin and hepatitis B vaccination that have been developed to prevent vertical transmission^6^^–^^10^ have widely been reported to be effective.^11^^–^^16^ Neonatal immunization was introduced to the Hong Kong Special Administrative Region (SAR), China, in 1983^1^ and was selectively applied to neonates born to mothers found to be carrying the hepatitis B surface antigen (HBsAg) on routine antenatal screening.^17^ In November 1988, neonatal HBV vaccination has become universal irrespective of maternal HBsAg status.^18^^,^^19^ Since then, vaccination has been readily available from general practitioners and nongovernmental organizations.^17^

In Hong Kong SAR, pregnant women receiving antenatal care constitute the only social group that undergoes routine HBsAg screening. We reviewed published reports of the prevalence of maternal HBsAg carriage in the area and found that, in the prevaccination era, it was 6.6% in 1976^20^ and 7.4% between 1981 and 1983.^1^ In 1996, it was 10.0% and, between 1998 and 2001, it was 8.0%.^21^ At our hospital, it was 10.1% in women who gave birth between 1998 and 2008.^22^ In our latest survey in 2010, the prevalence was 9.1% overall and 4.8% in women who had undergone HBV vaccination.^23^ Since the obstetric population is a low-risk group compared to those with traditional risk factors for acquiring HBV infection in adulthood, such as intravenous drug users and men who have sex with men, pregnant women can be taken as surrogates for the population at large. Consequently, the 9.0–10.0% prevalence of HBsAg carriage observed in these women suggests that Hong Kong SAR remains a high-endemicity area according to the World Health Organization’s definition; despite more than two decades of universal neonatal vaccination.^13^^,^^15^^–^^17^

Literature reports suggest that immunity conferred by the vaccine may not always be life-long. One study in Taiwan, China, reported that 1.3% of 2- to 6-year-olds who had undergone complete vaccination were HBsAg-positive,^24^ whereas another reported that 1.3–3.5% of immunized infants became HBsAg carriers 15 years later.^25^ Furthermore, in Alaska, United States of America, a precipitous decline in antibody titre was observed around the age of 15 years in children vaccinated at a very young age^26^ and anamnestic responses to booster vaccinations were absent in half of 15-year-olds vaccinated at birth.^12^ Even among fully vaccinated individuals in Taiwan, the seropositivity rate for antibody to HBsAg declined from 100.0% at the age of 2 years to 75.0% at the age of 6 years.^24^ In addition, 22.9% of vaccinated children in Israel had undetectable antibody levels, irrespective of gestational age, birth weight or parental origin.^27^ Indeed, it has been recommended that a single booster dose should be given 10 years after primary vaccination because protection was estimated to last between 7.5 and 10.5 years.^28^

A recent study in Pakistan found that the HBV infection rate rose from 13.4% among individuals aged 11 to 20 years to 34.9% among those aged 21 to 30 years.^29^ We observed recently that the prevalence of HBV infection in university students in Hong Kong SAR, increased with age: it was 0.9%, 2.3%, 4.3% and 5.5% in those aged ≤ 18, 19, 20 and ≥ 21 years, respectively.^30^ The prevalence also increased with age among women who underwent antenatal screening at our hospital: it was 2.5%, 2.7%, 8.8% and 8.0% in those aged ≤ 16, 17, 18 and 19 years, respectively.^31^ These findings suggest that immunity against HBV infection wanes in late adolescence, which could explain the persistently high prevalence of HBsAg carriage we observed in pregnant women. However, the situation in Hong Kong SAR is complicated by the fact that, since the return of sovereignty to China in 1997, there has been a steady influx of immigrants from the mainland, where the prevalence of chronic HBV infection ranged from 4.5% to 17.9% before HBV vaccination was introduced in 1992.^32^ Consequently, there was a small but steady addition of infected individuals to the pool of fertile women in Hong Kong SAR. Nevertheless, the extent to which these women contributed to the sustained high prevalence of maternal HBV infection in the region remains unclear.^17^^,^^21^^–^^23^ We hypothesized that the main reason for the current high prevalence of maternal HBsAg carriage was the progressive waning of vaccine-conferred immunity against HBV infection in late adolescence. Therefore, we re-examined the age-specific prevalence of positive antenatal HBsAg screening test results in pregnant women who had been vaccinated to determine if and when the age-specific prevalence of HBsAg carriage increases during adolescence.

## Methods

Our hospital department is one of the eight public obstetric units in Hong Kong SAR that provide free obstetric care to local residents. We serve a population of 1.2 million, which comprises one seventh of the region’s population, and, as the hospital is a tertiary referral centre, we accept referrals from throughout Hong Kong SAR. Our annual delivery rate of 7000 infants is the highest among the eight units. Obstetric management is protocol-driven and includes routine antenatal screening for HBsAg and immunity against rubella, in accordance with official local health policy. The results of investigations and pregnancy outcomes are recorded in a computerized database that covers all public hospitals in Hong Kong SAR. Data entry is performed by trained midwives and obstetricians and is doubled-checked after delivery. To determine whether and when a transition to the current high prevalence of HBV infection occurred during adolescence, we performed a retrospective cohort study of the age-specific prevalence of HBsAg carriage in mothers aged 25 years or younger at delivery who were born in Hong Kong SAR and who were managed between 1998 and 2011. We used data from our obstetric database, which has been previously validated.^22^ The study was approved by the Joint CUHK-NTEC Clinical Research Ethics Board. 

First, we analysed the age-specific prevalence of HBsAg carriage in the entire group. We investigated the effect on prevalence of the mother’s year of birth (i.e. before or in or after 1984, when HBV vaccination became available) and parity (i.e. nulliparity or multiparity) and identified the age at which the transition from a low to a high prevalence occurred. Subsequently, we performed further analyses based on the age of transition identified and, by taking into account the influence of being born before or in or after 1984, we investigated the effect of vaccination on prevalence below and above the age of transition. Statistical analyses were performed using the *χ^2^* test and odds ratios (ORs) and 95% confidence intervals (CIs) were calculated as appropriate. Correlations between prevalence and age were evaluated using Spearman’s correlation coefficient. Multiple logistic regression analysis was used to determine whether the prevalence was significantly influenced by parity, maternal birth before the implementation of HBV immunization or maternal age above or below the age of transition. Calculations were performed using SPSS Statistics 20 (IBM, Armonk, United States of America).

## Results

**Fig. 1:**
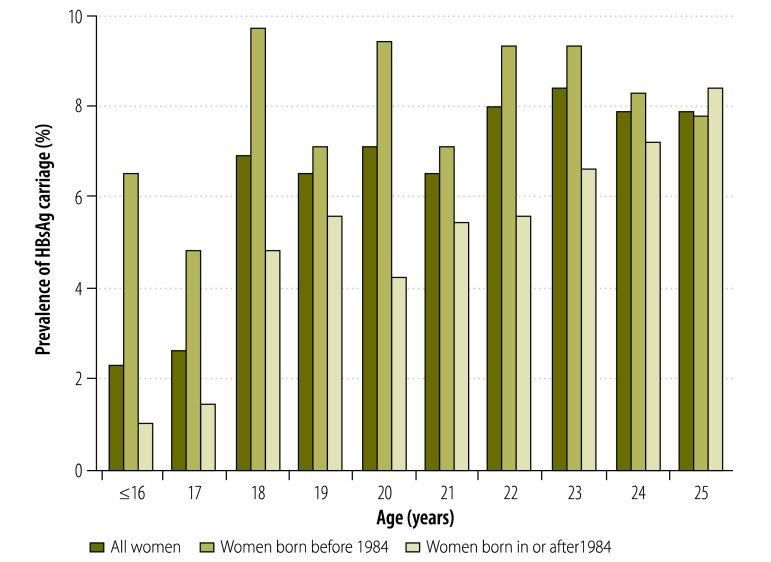
Prevalence of hepatitis B surface antigen carriage in young pregnant women, by age at parturition and year of birth, Hong Kong Special Administrative Region, China, 1998–2011

Of the 93 306 women who gave birth between 1998 and 2011 at our hospital, 10 808 (11.6%) were aged 25 years or younger. The overall prevalence of HBsAg carriage in these young women was 7.5%. As only 129 were aged 16 years or younger, they were grouped together for the analysis. The age-specific prevalence of HBsAg carriage is shown in Fig. 1. There was a significant difference between age groups (*P* = 0.020) and a positive correlation between age and prevalence (*P* = 0.006). The age-specific prevalence for women who were born before and in or after 1984 is also shown in Fig. 1 and in Table 1. For those born before 1984, there was no significant difference between age groups (*P* = 0.558) and no correlation between age and prevalence (*P* = 0.666). For those born in or after 1984, there was a significant difference between age groups (*P* = 0.018) and a significant positive correlation with age (*P* < 0.001).

HBsAg: hepatitis B surface antigen.

**Table 1:** Carriage of hepatitis B surface antigen by pregnant women, by age at parturition and year of their birth, Hong Kong Special Administrative Region, China, 1998–2011

Age at parturition (years)	No. of women			Prevalence of HBsAg carriage in women, no. (%)		Likelihood of HBsAg carriage by a woman born in or after 1984, OR (95% CI)
	born in or after 1984^a^	born before 1984^a^		born in or after 1984^a^	born before 1984^a^	
≤ 16	98	31		1 (1.0)	2 (6.5)	0.149 (0.013–1.708)
17	145	84		2 (1.4)	4 (4.8)	0.280 (0.050–1.561)
18	248	185		12 (4.8)	18 (9.7)	0.472 (0.221–1.006)
19	301	378		17 (5.6)	27 (7.1)	0.778 (0.416–1.456)
20	406	498		17 (4.2)	47 (9.4)	0.419 (0.237–0.742)
21	409	644		22 (5.4)	46 (7.1)	0.739 (0.438–1.248)
22	500	882		28 (5.6)	82 (9.3)	0.579 (0.371–0.902)
23	546	1162		36 (6.6)	108 (9.3)	0.689 (0.466–1.019)
24	545	1405		39 (7.2)	116 (8.3)	0.856 (0.587–1.249)
25	491	1850		41 (8.4)	145 (7.8)	1.071 (0.746–1.539)
**Total**	**3689**	**7119**		**215 (5.8) **	**595 (8.4) **	**0.679 (0.578–0.797)**

CI: confidence interval; HBsAg: hepatitis B surface antigen; OR: odds ratio.

^a^ Neonatal vaccination against the hepatitis B virus became available in 1984.

The most marked increase in prevalence occurred around the age of 18 years in women born both before 1984 and in or after 1984: there was a twofold and a greater than threefold increase between the ages of 17 and 18 years in the two groups, respectively. Therefore, a cut-off age of 18 years was adopted for the analysis of the effect of vaccine availability on the prevalence of HBsAg carriage (Table 2). The prevalence was found to be significantly lower in women born in or after 1984 than in those born before 1984: 5.8% versus 8.4%, respectively (*P* < 0.001). The figure was significantly lower in women born in or after 1984 among both those aged under 18 years (1.2% versus 5.2% for the two birth year groups, respectively; *P* = 0.025) and those aged 18 years or more (6.2% versus 8.4%, respectively; *P* < 0.001). However, the difference in prevalence between women aged under 18 years and those aged 18 years or more was significant only for those born in or after 1984 (1.2% versus 6.2%, respectively; *P* = 0.002). 

**Table 2:** Influence of being older than 18 years at parturition and being born before 1984 on hepatitis B surface antigen carriage by pregnant women, Hong Kong Special Administrative Region, China, 1998–2011

Women’s year of birth	Prevalence of HBsAg carriage (%)			Likelihood of HBsAg carriage by a woman aged < 18 years at parturition, OR (95% CI)
	All women	Women aged < 18 years at parturition	Women aged ≥ 18 years at parturition	
All	NA	2.5	7.7	0.311 (0.160–0.604)
≥ 1984	5.8	1.2	6.2	0.191 (0.061–0.600)
< 1984	8.4	5.2	8.4	0.600 (0.262–1.370)
OR (95% CI)^a^	0.679 (0.578–0.797)	0.227 (0.056–0.925)	0.714 (0.607–0.840)	NA

CI: confidence interval; HBsAg: hepatitis B surface antigen; NA: not applicable; OR: odds ratio.

^a^ Likelihood of hepatitis B surface antigen carriage by a woman born in or after 1984, when neonatal vaccination against the hepatitis B virus became available.

When the effect of parity was analysed, the only significant difference in prevalence found was between nulliparas born in or after 1984 and those born before 1984: 5.6% versus 8.2%, respectively; *P* < 0.001, Table 3). There was no significant difference in multiparas between those born before or in or after 1984 and no difference between nulliparas and multiparas in either birth year group.

**Table 3:** Influence of parity and being born before 1984 on hepatitis B surface antigen carriage by pregnant women, Hong Kong Special Administrative Region, China, 1998–2011

Women’s year of birth	Prevalence of HBsAg carriage (%)		Likelihood of HBsAg carriage by a nullipara, OR (95% CI)
	Nulliparas	Multiparas	
All	7.3	8.2	0.879 (0.743–1.038)
≥ 1984	5.6	6.7	0.824 (0.591–1.148)
< 1984	8.2	8.8	0.923 (0.760–1.120)
OR (95% CI)^a^	0.655 (0.553–0.799)	0.745 (0.532–1.044)	NA

CI: confidence interval; HBsAg: hepatitis B surface antigen; NA: not applicable; OR: odds ratio.

^a^ Likelihood of hepatitis B surface antigen carriage by a woman born in or after 1984, when neonatal vaccination against the hepatitis B virus became available.

The relative effect on the prevalence of HBsAg carriage of the year of the mother’s birth and the mother’s age at parturition was evaluated by multiple logistic regression analysis, with parity as a confounder. Being aged 18 years or more had a greater effect on prevalence (adjusted OR, aOR: 2.80; 95% CI: 1.46–5.47) than being born before 1984 (aOR: 1.42; 95% CI: 1.21–1.67). The effect of parity was not significant.

## Discussion

The long-term protection provided by a vaccine can be assessed from the anamnestic response to a booster dose, the infection rate in the vaccinated population, in vitro tests of B- and T-cell activity and seroepidemiological studies. The HBV vaccine is thought to induce life-long immunoprotection^11^^,^^13^^,^^14^ and the latest review suggests there is no need for a booster dose in immunologically competent persons.^33^ This view is supported by data showing that 97.0% of children demonstrated an anamnestic response to a booster dose 10 years after infancy vaccination, even if there was only a protective antibody concentration in 64.0% of the children.^34^ Nevertheless, other studies reported that effective protection lasted only 15 to 20 years.^12^^,^^25^^,^^26^^,^^35^^,^^36^ Even among fully vaccinated children, the seropositivity rate for antibody to HBsAg has been reported to decline.^24^ In one Israeli study, the proportion of children with an undetectable antibody level varied with the interval between vaccination and testing: it was 36.1%, 20.0% and 14.6% in children aged 5 to 8 years, 2.5 to 5 years and 1 to 2.5 years, respectively.^27^ The loss of protection appears to spare no ethnic group. In Micronesians vaccinated at birth, 8% showed evidence of past HBV infection 15 years later, although none had a chronic infection.^37^ Moreover, only 7.3% of uninfected individuals tested positive for antibody and only 47.9% of the remaining individuals had an anamnestic response. Among Australian Aboriginal adolescents, evidence of an active or past HBV infection was found in 11.0% and 19.0%, respectively, despite complete HBV vaccination in infancy.^38^ In Taiwan, vaccine failure, which occurred at a rate of 33.3–51.4%, was a major cause of hepatocellular carcinoma: the rate of the disease only declined from 0.54 to 0.20 per 100 000 children aged 6 to 14 years from before to after the introduction of the vaccination programme. Paradoxically, the risk of the disease was higher in HBV carrier children born after the vaccination programme (risk ratio: 2.3 to 4.5).^39^ Thus, it is questionable whether the HBV vaccine offers long-term protection.

In our study, we observed an age-related increase in the prevalence of HBV infection in women undergoing routine antenatal screening. Although we could not be absolutely certain all these women had completed a course of HBV vaccination, it is likely they had for the following reasons. In addition to the neonatal vaccination programme in Hong Kong SAR, catch-up vaccination was also given to all school students born before 1983.^40^ Moreover, in July 1992 the vaccination programme was extended to cover all children born between January 1986 and November 1988 so that, by the end of the 1990s, all children aged 13 years and younger and residing in Hong Kong SAR, irrespective of place of birth, would have been immunized.^41^ Finally, a supplemental HBV vaccination programme was launched in the 1998 to 1999 school year for primary 6 students who had not received or completed the three-dose regimen – the aim was to protect all children before they became sexually active.^42^ Indeed, our data indicate that the vaccine conferred a protective effect because the prevalence of HBV infection detected by antenatal screening was significantly lower in women born in or after 1984 than in those born before 1984. However, the prevalence of HBsAg carriage increased with age even in these protected women. In Hong Kong SAR, the prevalence of HBV infection before the introduction of vaccination was 6.6% in 1976^20^ and 7.4% in 1981 to 1983.^1^ Thus, the 6.9% prevalence of HBsAg carriage we observed in 18-year-old pregnant women born in or after 1984 was comparable to the 1976 figure and the 8.0% prevalence we observed in 22-year-old women exceeded the figure for 1981 to 1983. Moreover, the 8.4% prevalence we observed in 23-year-old women was the same as in the locally born, resident, adult population in 1996.^17^ Consequently, our observation of a substantial infection rate in a vaccinated population, taken together with other similar reports in the literature,^25^^,^^26^^,^^35^^,^^36^^,^^38^^,^^43^ provides compelling evidence that the HBV vaccine does not provide long-term protection.

Before mass HBV vaccination was introduced, a study found that the rate of HBV infection increased from 1.7% among children aged 3 to 5 years to 4.5% among teenagers aged 17 to 19 years,^44^ which suggests that horizontal transmission is important during the transition to adulthood. In addition to transmission through sexual intercourse and high-risk behaviours,^45^ such as the sharing of needles among heroin addicts, horizontal HBV transmission can occur within the family and community in high-endemicity areas because HBV is present in body fluids such as saliva and urine.^43^^,^^46^ One study found that 17.5% of offspring were HBsAg-positive if either parent was HBsAg-positive compared with 1.5% if both parents were HBsAg-negative^43^ and that the presence of an HBV-infected family member was a risk factor at the individual level. This is important in Asia where eating and living with in-laws after marriage is common. It could be that exposure of individuals with declining immunity to infection was one reason for the increase in the prevalence of HBsAg carriage from 1.9% in adolescents to 4.9% in those aged 20 to 30 years reported in the Republic of Korea^43^ and for our earlier observation that the prevalence of HBV infection rose rapidly among teenagers in Hong Kong SAR.^30^^,^^31^

The logical way to reduce the risk of horizontal transmission to individuals with waning immunity is to give a booster dose of HBV vaccine. However, there is no consensus on the necessity of booster doses after neonatal immunization.^12^ More than a decade ago it was recommended that 12-year-old children should receive a single booster dose 10 years after primary triple-dose vaccination because protection was estimated to last only 7.5 to 10.5 years.^28^ Indeed, one study found that the geometric mean decay in the titre of antibody to HBsAg between the ages of 7 and 16 years in children who did not receive booster doses was 20.0% per year.^47^ Moreover, there is growing evidence that the antibody titre declines with age, especially after the age of 15 years.^12^^,^^24^^–^^27^^,^^47^ The latest guidelines of the Steering Committee for the Prevention and Control of Infectious Diseases in Asia recommend a booster dose of HBV vaccine: (i) 10 to 15 years after primary vaccination in Asian populations where the virus is highly endemic and where it is not feasible to monitor antibody levels; (ii) in immunocompromised patients when the antibody titre falls to below10 mIU/mL; and (iii) in health-care workers.^48^ A booster dose was also suggested for individuals who have a poor response to the vaccine and for high-risk adolescents.^49^ Nevertheless, a single booster dose may not be sufficient as it has been reported that 2.7% to 3.3% of children remained antibody-negative after the booster,^25^^,^^34^ probably because it did not induce an immune response in healthy adolescents who had an undetectable antibody titre. Responses can be impaired by factors such as ethnicity and substance use.^49^

There is no consensus on the timing of the first booster dose after neonatal vaccination. Reports of declines in antibody titre and protection 10 to 15 years after childhood vaccination,^12^^,^^23^^,^^25^^–^^27^^,^^47^ of a precipitous decline in antibody titre at the age of 15 years^26^ and of absent anamnestic responses in half of 15-year-old children vaccinated at birth^12^^,^^34^ all suggest that the immunoprotection induced by the vaccine cannot be guaranteed beyond 16 years of age. In China, the HBsAg seropositivity rate has been reported to increase from 0.3% in individuals aged 15 to 17 years to 1.4% in those aged 18 to 19 years, eventually reaching 3.0% in those aged 24 years.^50^ Overall, the evidence – especially our finding that there was a transition to a high prevalence of HBV infection at 18 years of age – supports the recommendation that a booster dose should be given to all adolescents aged between 16 and 17 years.^27^^,^^47^^,^^48^ At present, neither Hong Kong SAR nor any Asian country recommends or gives a routine booster to all adolescents.

Although our study population was limited by the fact that our hospital serves only one seventh of the total population of Hong Kong SAR, the district served comprises mostly new towns that have been developed in the past 20 years to accommodate younger people with new families moving from other districts. Consequently, the young pregnant women in our study are likely to be representative of those who live in other districts and, therefore, of the local female population.

In conclusion, our study found that the prevalence of maternal HBV infection in Hong Kong SAR increased from a low to a high rate around the age of 18 years. Above that age, the prevalence of HBsAg carriage in young mothers who should have been protected by neonatal HBV vaccination returned to that observed in 1976 before the vaccination programme was implemented. Since the pregnant women in our study were not at a high risk of acquiring an HBV infection through horizontal transmission, our findings challenge the view that HBV vaccination confers life-long protection. Future studies should investigate the timing and extent of waning immunity against HBV infection with age, the anamnestic response rate and the most cost-effective way of maintaining immunity in adolescents vaccinated in infancy.
